# A Phenological Model for Olive (*Olea europaea* L. var *europaea*) Growing in Italy

**DOI:** 10.3390/plants10061115

**Published:** 2021-05-31

**Authors:** Arianna Di Paola, Maria Vincenza Chiriacò, Francesco Di Paola, Giovanni Nieddu

**Affiliations:** 1 Institute for BioEconomy, National Research Council of Italy (IBE-CNR), 00100 Rome, Italy; 2 Impacts on Agriculture, Forests and Ecosystem Services Division, Centro Euro-Mediterraneo sui Cambiamenti Climatici (CMCC), 01100 Viterbo, Italy; mariavincenza.chiriaco@cmcc.it; 3 Institute of Methodologies for Environmental Analysis, National Research Council of Italy (IMAA-CNR), 85050 Tito, Italy; francesco.dipaola@imaa.cnr.it; 4 Department of Agricultural Sciences, Sassari University, 07100 Sassari, Italy; gnieddu@uniss.it

**Keywords:** olive, phenological model, developmental rates, agrometeorology

## Abstract

The calibration of a reliable phenological model for olive grown in areas characterized by great environmental heterogeneity, like Italy, where many varieties exist, is challenging and often suffers from a lack of observations, especially on budbreak. In this study, we used a database encompassing many phenological events from different olive varieties, years, and sites scattered all over Italy to identify the phases in which site-enlarged developmental rates can be well regressed against air temperature (Developmental Rate function, *DR*) by testing both linear and nonlinear functions. A K-fold cross-validation (KfCV) was carried out to evaluate the ability of *DR* functions to predict phenological development. The cross-validation showed that the phases ranging from budbreak (BBCH 01 and 07) to flowering (BBCH 61 and 65) and from the beginning of flowering (BBCH 51) to flowering can be simulated with high accuracy (*r*^2^ = 0.93–0.96; RMSE = 3.9–6.6 days) with no appreciable difference among linear and nonlinear functions. Thus, the resulting *DRs* represent a simple yet reliable tool for regional phenological simulations for these phases in Italy, paving the way for a reverse modeling approach aimed at reconstructing the budbreak dates. By contrast, and despite a large number of phases explored, no appreciable results were obtained on other phases, suggesting possible interplays of different drivers that need to be further investigated.

## 1. Introduction

Olive (*Olea europaea*, L.) is a long-lived, drought-tolerant species strongly adapted to the Mediterranean climate [1], where it is counted among the oldest and most important tree crop species [2]. In this region, temperatures lower than −8 °C limit its northward distribution, whereas annual rainfalls lower than 350 mm y^−1^ limit its distribution in arid regions [1,3].

Temperature is the most influencing driver controlling the phenology of olive trees, especially flowering, for which a positive, mostly linear, relationship has been confirmed by a large number of studies [4,5,6,7,8,9]. Temperature also controls the induction of winter rest and the subsequent vegetative onset [10], although the underlying mechanism is still poorly understood since the related phenological processes are difficult to observe. Several studies suggest that the olive tree requires a chilling period, known as *endodormancy*, to break the winter rest but little is known about its specific time span and the exact amount of chilling required [11,12]. Moreover, in different bioclimatic areas, large variability in the likely chilling period could emerge [11]. 

Other drivers reported in literature are genotypic diversity [13], latitude [14], topography [6,15,16] and rainfall [9,15]. However, it is worth noting that in a multi-environment trial [13], the genotype by environment interaction explained less than 10% of the observed variance in flowering dates, with genotypic diversity mostly affecting the quality of flowering. Lastly, the photoperiod, a major driver of spring phenology and flowering for many plant species [17,18], has a rather disputed role on the phenology of olive, as in some cases it did not show remarkable effects [7,14,19], while in others it improved the prediction of flowering when used as a threshold to start cumulating temperatures in a thermal sum model [20,21].

Due to the strong ecological and economic relevance of olive groves, a large number of phenological models have been developed for olive in recent decades. Phenological models are essential tools for many crop management issues, as they allow for scheduling crop practices, reducing climate risks, optimizing external resources, and enhancing pest and disease control [22,23,24]. For instance, the development and spread of many flower- and seed-eating pests depend on the flowering date or the period of seed setting of their host plant [25], making the forecast of flowering fundamental for pest management [26]. Moreover, phenology is considered a bio-indicator of climate changes and phenological models have also been widely used to assess them in different areas of the Mediterranean region [3,7,27].

Phenological models for olive are generally statistical regressions of airborne pollen data to some meteorological variables [9,14,20,21,28] or a derivation of a thermal sum model applied on a local scale to predict the day of flowering [4,7,11,19,29]. Several thermal sum models include a chilling period and the estimation of the starting date to cumulate the temperatures until flowering (alternatively, the starting date most widely used in the northern hemisphere is 1 January). However, this topic still remains a challenge, especially on a large scale, as the endodormancy is not easily identifiable and the budbreak is rarely observed. 

On a large scale, the applicability of phenological models is further hampered by the large variability in the thermal availability over the growing season [30]. Few studies have been scaled up from local to regional scale [9,20,31] but they relied solely on the prediction of flowering, except for the model implemented in [31] that is currently used by the Italian Council for Agricultural Research and Economics (CREA, Rome, Italy) to forecast many phenological events of different plant species, including olive (phenological bulletins are available at http://cma.entecra.it/iphen/bollettini.asp, last access: 28 May 2021). The main advantage of this model relies on the prediction of many olive phenological events (i.e., not only flowering). However, due to the heterogeneity of olive varieties and of the environmental conditions at national level, results need to be corrected with in-season local phenological observations.

Indeed, the heterogeneity in crop varieties, geography, and environment that characterize Italy makes the development of a reliable phenological model challenging. However, we argued that if the temperature is the most influencing driver controlling the phenology of olive trees, then an approach that explores its predictive power over many phases embracing a heterogenic pool of data could help overcome the problems arising from the environmental and genotypic diversity.

The present work aimed to ascertain if the above hypothesis is correct and verify whether it is possible to calibrate a simple, generalized, large-scale phenological model for olive growing in Italy based on air temperature. Such a model would allow for regional applications with a minimal input of data, under a wide range of environmental conditions, and/or where the reference to a single variety could be reductive. To this end, we used the data collected within the project PHENAGRI (1996–2003) [32], which has the valuable peculiarity of including observations of many phenological events, including budbreak, from different olive varieties, years, and experimental sites scattered all over Italy, to identify the phases whose developmental rates could be well regressed by a function of air temperatures, testing both linear and nonlinear curves. Moreover, a possible improvement from the use of daylength as a predictor variable was also tested. Single models were tested phase by phase with a K-fold cross-validation (KfCV) [33]. KfCV is a well-established method for model evaluation where each sample has the opportunity of being tested one time as well as serving as training data in the remaining K−1 times, allowing the full exploitation of the data set and, thus, making the evaluation more reliable. Highly performing phases were chosen to embody the phenological model and findings were discussed in the light of the current knowledge of olive phenology.

## 2. Materials and Methods

### 2.1. Data Source

We used phenological field observations collected within the project PHENAGRI (1996–2003) [32]. In such a project, a large number of field observations on both weather and phenological development of olive trees were collected from seven experimental sites widely spread over Italy (Table 1). Weather data consisted of daily records from in situ meteorological stations (when present) or from the nearest reference station belonging to the national networks of CREA or to the Air Force Met service (AFM, Roma, Italy).

The dataset includes observations on 17 olive varieties representative of the different geographical areas in which the experimental sites were located. Three varieties (Carolea, Coratina, Picholine) are common to all locations. For each experimental site and olive variety, four shoots were chosen on four different plants to take the observations. Winter rest and vegetative onset were periodically monitored by looking at both the apical and lateral buds. The subsequent phenological events were observed over the same shoots. The date of a single phenological event was defined as the median between the dates recorded on each plant. Field surveys to monitor plants’ phenology were performed with a variable frequency depending on the ongoing phenological phase: every 4 days during flowering and every 7 days during the remaining phases Further details on the operational protocol used for the collection of data adopted by the PHENAGRI project are reported in [34].

**Figure 1:**
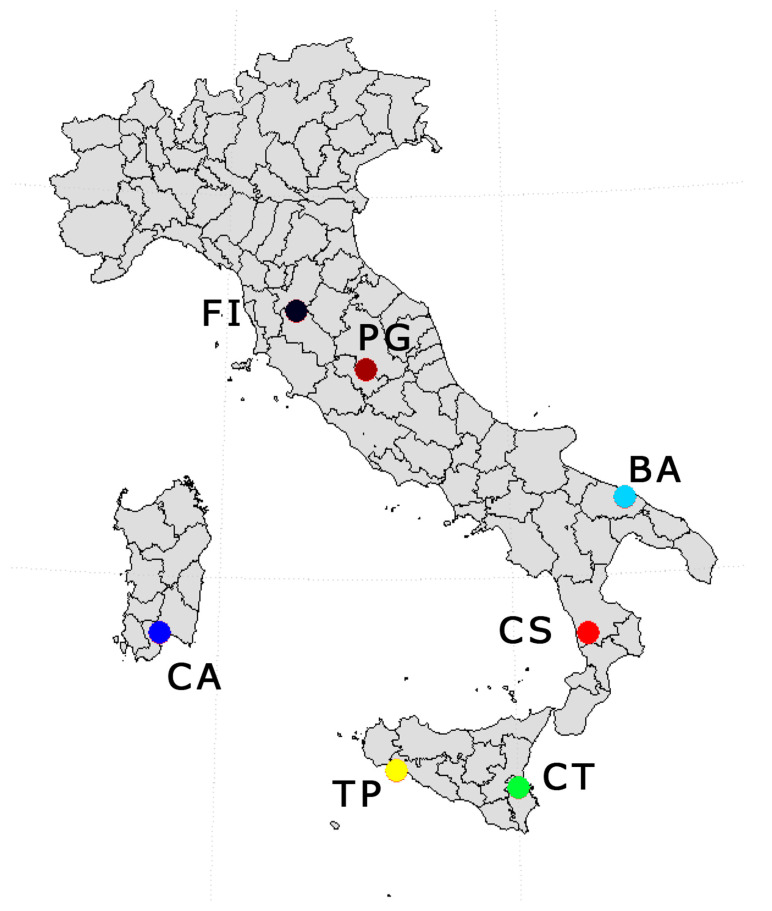
Geographical distribution of the experimental sites in the PHENAGRI project. The abbreviations indicate the provinces where the experimental fields were located.

**Figure 2:**
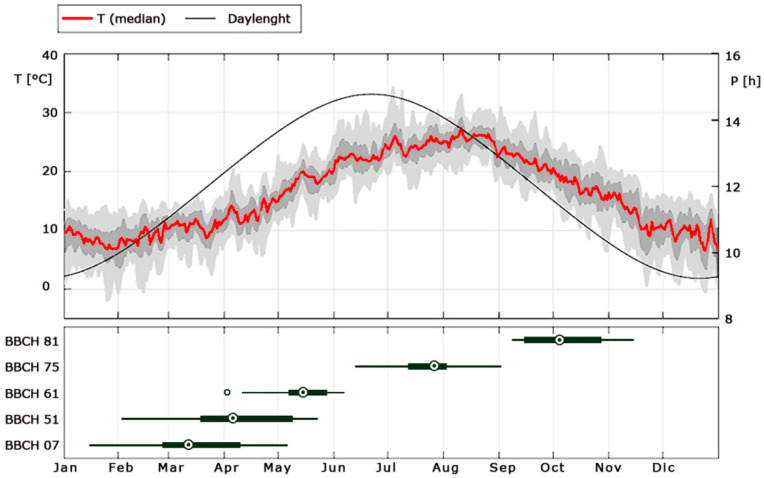
Upper panel: average daily temperatures (red line as median, dark grey shadows as interquartile range, light grey shadow as 5th to 95th percentile) and daylength (black line) between experimental sites (Table 1) during the period 1997–1999. Bottom panel: distributions of phenological observations (DOY) for the representative events BBCH 07, 51, 61, 75, and 81 (definitions in Table 1) among experimental sites, period 1997–1999. In the boxplot the central dot is the median, the boxes are the 25th and 75th percentiles, whiskers extend to the 10th and 90th percentiles. Empty dot: outlier.

The observed phenological events, reported in BBCH centesimal scale [35], are summarized in Table 2. Figure 1 shows the geographical distribution of the experimental sites, while Figure 2 shows the variability of daily temperatures among sites experienced over the period 1997–1999 along with the observed distributions of some representative phenological events expressed as the day of the year (DOY) when they occurred. 

### 2.2. Developmental Rate (DR) Function 

A versatile way to model plant development is to regress a phase maturity rate (the reciprocal of the phase time length) against the mean value(s) of the predictor variable(s) experienced during that phase [36]. Here, we call the resulting equation (eq.) Developmental Rate (*DR*) function, regardless if linear or not, while we refer to *phase* as the period between two distinct *phenological events*.

#### 2.2.1. Linear DR Function

As earlier suggested by [37] for cereal crops or [38] for insect populations, a *DR* function for a given phase could be assumed as a linear function of mean air temperature:

*DR = a + bT* (1)

where *DR* is the developmental rate, i.e., the reciprocal of the phase time length [d^−1^], *T* is the mean air temperature experienced during the phase [°C], *a* is the intercept [d^−1^], and *b* is the slope [°C^−1^d^−1^] determined by the linear regression.

The intersection of the linear *DR* function with the *x*-axis returns the value for the base temperature (*T*_0_ *= −a/b*, [37,38]) that represents the critical temperature below which phase development is assumed to be nil since the *DR* would assume negative values. Under the assumption of linearity, the reciprocal of the slope corresponds to the thermal constant, or growing degree-days’ requirement of the well-known thermal sum model, and the base temperature is the threshold above which degree-days are cumulated [38]. Similarly, following the approach adopted by [30] and [37], whenever an additional linear relation exists among *DR* and daylength, a multiple linear regression can be considered as:

*DR = a + bT + cP* (2)

where *P* is the mean daylength during the phase expressed in hours [h] and *a*, *b,* and *c* are the coefficients of the multiple linear regression. Formally, even in the case of multiple linear regression, the developmental rate stops when the temperature or daylength falls below critical values (*T*_0*m*_ and *P*_0*m*_, respectively, where the subscript m stands for “multiple”). The values of *T*_0*m*_ and *P*_0*m*_ can be retrieved by the intersection of the linear *DR* function (projected on a two-dimensional scatter plot) with the corresponding abscissa. Examples of how simple and multiple linear regressions behave are shown in Figures S1 and S2, respectively. 

#### 2.2.2. Nonlinear DR Function

Many different nonlinear functions have been used in literature (e.g., Poisson equation [39], second-degree polynomial [40], sigmoidal function [41]) to represent nonlinear trends of plant developmental rates sometimes observed over the full range of tolerable temperature conditions to which the plant is exposed. 

In the present work, a second-degree polynomial was proposed, expressed in the form of

*DR = a + bT + cT^2^* (3)

The polynomial function expressed in Equation (3) allows us to represent curvilinear trends of developmental rates against temperature, if any, and has the advantage of being linear in terms of the unknown coefficient. As for linear functions, the developmental rate stops when temperatures fall below critical values (*T_p_*_1_ and *T_p_*_2_). The estimation of critical values is given by the general formula to find the intersection of a parabola with the *x*-axis. An example of how the polynomial regression behaves is shown in Figure S3.

#### 2.2.3. Using DRs to Simulate Olive Phenology

Since the developmental rate is the inverse of a given time phase length, by definition, its integration over the phase time length must return 1. For this reason, the prediction of a given phase time length (*S*) could be computed with only the inputs of the starting date of the phenological phase and the daily predictor values as follows:

*Σ^S^_j_*_= 1_*DR_j_* = 1 (4)

where *DRj* is the daily rate computed for the *j*-day according to:

- Equation (1) when a simple linear *DR* is adopted:

*DR_j_ = a + bT_j_*  (if *T_j_* > *T*_0_) (5)

*DR_j_* = 0   (if *T_jn_ < T*_0_) (6)

where *Tj* is the daily mean air temperature of the *j*-day;

- Equation (2) when both temperature and daylength are explanatory variables:

*DR_j_* = *a* + *bT_j_* + *cP_j_*  (if *T_j_* > *T*_0*m*_ and *P_j_* > *P*_0*m*_) (7)

*DR_j_* = 0   (if *T_j_ < T*_0*m*_ or *P_j_ < P*_0*m*_) (8)

where *Pj* is the daily mean daylength of the *j*-day; and

- Equation (3) when nonlinear *DR* is adopted as follows:

*DR_j_ = a + bT_j_ + cT_j_*^2^ (if *T_j_* > *T_p_*_1_ and *T_j_ < T*_*p*2_) (9)

*DR_j_* = 0   (if *T_j_ < T_p_*_1_ or *T_j_> T*_*p*2_). (10)

According to Equation (4), the end of the phase is achieved when the cumulative sum of the daily rates reaches 1 (*Summing Rates Method*, [38]). 

For annual crops, the starting date of the vegetative period corresponds to the sowing date, while for tree crops the starting date could correspond to the budbreak and should be observed in the field or estimated by a specific model. Lastly, the DOY at which the phase reaches its maturity can be easily deduced by summing the phase time length (*S*) to the DOY of the starting date.

### 2.3. K-Fold Cross-Validation (KfCV) and Final Model Calibration

**Figure 3:**
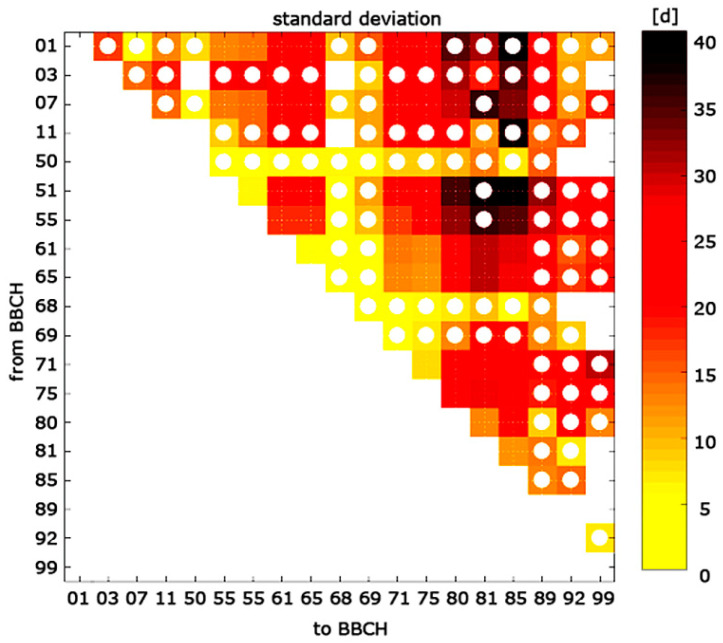
Standard deviation of olive phenological phases time length. *x*-axis: phenological events defining the beginning of a phase expressed in BBCH scale (definition in Table 2); *y*-axis: phenological events defining the end of a phase expressed in BBCH scale. Empty cells indicate the absence of data, white dot markers indicate phases discarded because of data embrace in less than 5 experimental sites.

From the whole database, counting approximately 1200 phenological observations, we computed the average developmental rates [d^−1^], average air temperature [°C], and daylength [h] experienced during all the possible phases resulting from pairwise combinations of phenological events, with a total of 171 phases inspected. Daylength was computed according to the FAO guideline [42] on a daily basis. Phases embracing data from less than five experimental sites were discarded to guarantee heterogeneity in the sample data, for a total of 48 phases selected (Figure 3). The average variability of the observed phases’ time lengths was described by computing the standard deviation for single phases.

K-fold cross-validation [33] was used on the selected phases to evaluate the ability of *DR* functions in predicting phenological development. According to this methodology, data of single phases (phase length, average air temperature, and daylength) are randomly mixed and partitioned into K-equal-parts, where each part is called *fold*. One fold is used as a validation subset, while the remaining folds are used as a training subset. A total of K rounds of training and validation are performed through an iteration that alternatively uses a different k-fold as validation subset and the remaining K-1 folds for the training procedure. In our case, K was fixed at 5. In each iteration, a *DR* function is regressed using ordinary least squares technique over the training subset. The obtained coefficients are then used to simulate phenological development according to Equation (4) using the predictor(s) data from the validation subset. Simulations, i.e., the phase time lengths, are finally compared with the observations from the validation subset using the coefficient of determination (*r*^2^, dimensionless), the Root Mean Square Error (RMSE, days), and Mean Bias Error (MBE, days) [43]. The RMSE represents the standard deviations of the model errors when the bias is null: the lower the values of RMSE, the higher the agreement of the model prediction with the observations. MBE indicates the average bias in the model predictions. The lower the values of MBE, the lower is the bias in the simulations. Moreover, since the initial random split of the data set into K groups may slightly affect the results, the KfCV was repeated N times (with N = 10) to remove potential biases and increase the model generalization. Figure S4 provides an example of training and validation over a single k-fold iteration. The overall ability of a *DR* function to predict the corresponding phase length is summarized through the ensemble mean of K × N runs [33]. Phases resulting with overall *r*^2^ higher than 0.8 and RMSE lower than 7 days were selected as cross-validated and subject to the final considerations on how the different kinds of *DR*s (i.e., simple, multiple, and nonlinear) perform and which ones can better embody the phenological model of olive. The adopted threshold of RMSE reflects the uncertainty of field measurements that could arise from surveys carried out on a weekly basis, as done under the PHENAGRI project [34].

After the cross-validation was completed, the best *DR* functions for single selected phases were calibrated using all the available data.

## 3. Results

Of the 48 selected phases, those broadly ranging from the budbreak (i.e., BBCH 01 and 07) to flowering (i.e., BBCH 61 and 65) had wide dispersions in the time phase length, as shown by the standard deviations (Figure 3). A large dispersion was also evident in phases ranging from BBCH 71 to 85. In general, the observed wide dispersion in budbreak to flowering was mostly due to the great variability in the DOY of budbreak (Figure 2, boxplot for BBCH 07), as the dates of flowering were rather synchronized around the middle of May (Figure 2, boxplot for BBCH 61). Similarly, great variability in the DOY of fruit maturity (Figure 2, boxplot for BBCH 81) seemed to explain the relatively high dispersion observed in the time phase length of the latter phases (Figure 3), namely, those broadly ranging from fruit development to fruit maturity. Again, Figure 2 shows that the variability in the DOY of the main phenological events (interquartile range of boxplots in Figure 2) reflects the variability of air temperature (grey shadows in Figure 2), which, in turn, over Italy is larger in winter and narrower in late spring.

**Figure 4:**
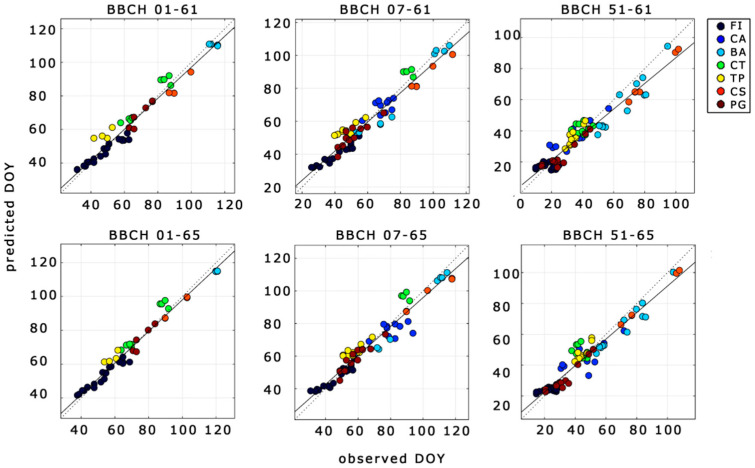
Simulated vs. observed phases time lengths for the cross-validated phases (subplots’ headline) using a linear DR function. Black line: least square line; dotted black line: 1:1 line. Statistical evaluation reported in Table 3 (Model of Equation (1)). Definition of BBCH in Table 2. Symbols in the legend refer to the location of the experimental site (Table 1).

Among selected phases, the KfCV showed meaningful results only for six phases (hereinafter, *cross-validated phases*) despite different types of *DR*s tested (i.e., Equations (1)–(3)). Cross-validated phases and related statistics are summarized in Table 3. Results showed that neither the introduction of daylength nor the use of polynomial function led to any substantial improvements to the results. Hence, the scatter plot between simulations and observations reported in Figure 4 only refers to simple, linear *DR*s.

Linear *DR* functions were able to simulate with high performance the phenological phases ranging from BBCH 01 and 07 to BBCH 61 and 65 (*r*^2^ = 0.93–0.95; RMSE = 4.7–5.7 days), good performance those from BBCH 51 to BBCH 61 and 65 (*r*^2^ = 0.93; RMSE = 5.7–6.6), while for the remaining phases no appreciable results were found. 

Overall, flowering can be predicted with high accuracy from BBCH 01 and 07 (*r*^2^= 0.93–0.96, RMSE = 3.9–5.6 days) and tolerable accuracy from BBCH 51 (*r*^2^ = 0.93, RMSE =5.7–6.6 days). Moreover, the RMSEs of the predicted phases listed in Table 3 were lower than the observed standard deviations of phases’ time length (Figure 3 and Table 3) and were also comparable with the observed intra-sites’ standard deviations of flowering, which ranged from 1.0 to 7.8 days (not shown). MBE denotes almost no bias (MSE < 1 day) from linear *DR*s for phases ranging from BBCH 01 and 07 to BBCH 61 and 65 and a moderate bias from nonlinear *DR*s over all the phases (MSE from ȡ2.89 to ȡ5.42 days) as well as from linear *DR* in BBCH 51–61 (MSE ranging from ȡ1 to ȡ2 days).

**Figure 5:**
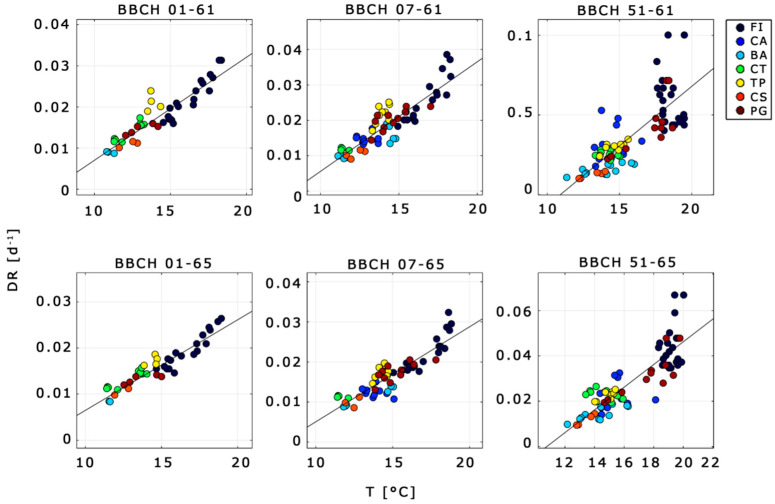
Calibration of *DR*s for cross-validated phases (subplots’ headline) using a linear DR function. Black lines: DR function. Statistical evaluation reported in Table 4. Definition of BBCH in Table 2. Symbols in the legend refer to the location of the experimental site (Table 1).

The final calibration of linear *DR*s for the cross-validated phases are shown in Figure 5, while the scores of coefficients and the related statistics are shown in Table 4.

When using all the available data, the temperature alone did explain up to 85–89% of the observed, site-enlarged variability in the phases’ time length, ranging from budbreak to beginning of flowering (BBCH 61), and 79–81% in the phases ranging from budbreak to full flowering (BBCH 65). The variability explained by temperature alone decreased to 62–74% in the remaining phases.

## 4. Discussion

By exploring a large phenological database, we were able to identify the phases where a *DR* function could predict olive phenological development with high performance. Indeed, the flowering event (BBCH 61 and 65) was simulated with high accuracy (*r*^2^ = 0.93–0.96; RMSE = 3.9–5.6 days, no bias) from budbreak (BBCH 01 and 07) and acceptable accuracy (*r*^2^ = 0.93; RMSE = 5.7–6.6 days, MBE < 2 days) from the beginning of inflorescence (BBCH 51) by simple, linear *DR* functions of temperature, regardless of the variety and/or geographical location. The model’s errors in predicting the date of flowering (3.9–5.6 days) from budbreak were comparable with the observed intra-sites’ variability (1–7.8 days), suggesting that further accuracy might be possible only by scaling down the model on a local scale and/or single variety. Furthermore, the errors on flowering were comparable with those found elsewhere [9,11,19,29,44], which ranged from approximately 3 to 8 days, depending on the experimental design. Therefore, the resulting calibrations for linear *DRs* represent a simple yet reliable tool for regional simulations of budbreak-flowering, allowing the possibility of a reverse modeling approach aimed at reconstructing the budbreak dates, rarely observed, starting from the event of flowering, which notoriously is the most observed phenological event. To our knowledge, this potential use is a novelty among olive phenological models. By contrast, no appreciable results were obtained in phases other than budbreak- and beginning of inflorescence-flowering, despite the large number of phases explored (171 from a pairwise combination, of which 48 had data from at least five experimental sites) and the use of different *DR* functions (i.e., linear and nonlinear). Indeed, the KfCV showed that neither the introduction of daylength nor the use of nonlinear *DR* functions led to any improvement in the results with respect to the linear *DR*, both in terms of performance and number of cross-validated phases.

The absence of significant results in phases ending at events other than flowering was consistent with and might explain the fact that almost all the existing phenological models for olive are focused solely on flowering. Moreover, phenological observations on events other than flowering, characterized by a higher variability (Figure 2), for instance, the budbreak, are rarely collected, limiting the possibility of exploring further phases. 

The absence of any improvement using nonlinear *DR* functions has already been found elsewhere [45,46] and it might suggest, in our case, the absence of nonlinear responses. The latter might have various explanations, one of which being the non-exposure of plants beyond their limits of tolerance. Again, it may happen that only a few points in the sample data show a relatively low developmental rate at high temperatures. If so, the nonlinear approximation may closely fit the training subset. However, when such regression is used in extrapolation, the scarce generalization of the calibration fails to predict the observations better than the other methods. Lastly, the average of temperatures over a long period may hide the occurrences of extremely high temperatures, if any. In all these cases, our findings suggest linear *DR* function as the most robust model providing results with tolerable error. 

Regarding the genotypic diversity, data on individual varieties were neither sufficient to test a model for a single variety nor to compare the phenology between varieties. Indeed, the study of genotypic diversity was outside the scope of the present article. However, if we look at the three olive varieties common to all the experimental sites (i.e., Carolea, Coratina, and Picholine), no difference seemed to arise between developmental rates over the cross-validated phases (Figure S5). Nevertheless, a larger amount of data for single varieties is required to draw a more robust conclusion.

Our approach was based on two underlying principles: First, the use of data collected under heterogeneous environmental conditions and from different olive varieties to overcome many local peculiarities to scale up the work from field to large scale, and second, the exploration of all the possible phases to identify those in which the calibrations of *DR*s, including the base temperature, which is usually extracted from literature (e.g., [4,15,19]), were better performing. By applying these principles, our approach allowed us to minimize the residual variability from drivers other than temperature, as those attributable to genetic diversity, microclimate, the proximity of the meteorological stations, and farming practices. The exploration of all possible phases to identify the suitable ones *a posteriori* was a novelty among olive phenological models and it allowed us to increase our knowledge on the phenology of olive tree. Indeed, our results confirmed the following points. (1) A strong phenological temperature response emerged in the broad phase, ranging from budbreak to flowering. Such a response is high enough to allow a unique site-enlarged calibration for the Italian domain based solely on air temperature. By contrast, in phases where a linear temperature response was weak or not present at all, the thermal sum model was baseless, justifying some perplexities raised on its low forecasting efficiency [47] and/or the need to differentiate calibrations among sites, as done in many studies [7,12,19,20]. A baseless thermal sum model might also explain the large variability in the heat requirements counted using a predetermined base temperature (i.e., not calibrated) observed among different sites, as shown in [14]. Moreover, the strong linear temperature response in the phase ranging from budbreak to flowering suggested the event of budbreak as the optimal date to start cumulating the heat requirements, usually fixed at 1 January or at the end of endodormancy (e.g., [19,20,31]), which is usually modelled without a validation since it is intrinsically hard to be observed. As a consequence, a model able to predict the budbreak date rather than the end of endodormancy might be linked to the presented model, allowing a better validation of the processes as well as broadening our current knowledge on the phenology of olive. (2) Over the Italian domain, olive trees show synchrony in flowering when the climate variability is relatively low (grey shadows in Figure 2, upper panel), but a great variability in the budbreak, when the climatic variability is relatively high. This can be well explained by a summing rates (thermal sum) model: At the budbreak, early varieties find relatively lower temperatures and grow relatively slower than the late ones, which, on the contrary, find relatively higher temperatures and, thus, grow a little faster. The same could hold for a single variety in different locations. Once again, it follows that a reliable calibration of the base temperature plays a fundamental role, as previously suggested by [48,49], but it requires a strong temperature response in developmental rates. (3) Olive developmental rates proved insensitive to daylength, as also reported before [7,14,19]. (4) The timing of phases beginning from, or after, flowering is mostly driven by variables other than temperature or by some of their interactions that still need to be further investigated to be implemented in a phenological model. In Figure S6 we showed the Pearson correlation matrix between phase time length and average temperature (left panel) and accumulated precipitation (right panel). In phases after flowering, the time lengths still had a relevant yet relatively weaker correlation with the average temperature compared to those observed from the budbreak to flowering, while accumulated precipitation showed an important correlation with phases’ time length in the period ranging from the beginning of fruit development to fruit maturity. The relationship between fruit development and water availability was already recognized in [50,51]. In particular, early ripening has been observed in years with limited precipitations and relatively warmer seasons. However, further investigations are required to obtain a predictive model on fruit development by considering multiple meteorological variables. 

Our work does feature some limitations that should be discussed. Firstly, phenological phases generally reflect some ecological, managerial, or even public health interests, such as periods when the plant is particularly vulnerable to external stressors, when it increases the demand for nutrients or releases pollen, causing some allergies. In olive, flowering is the most studied phenological event because olive pollen causes seasonal respiratory allergies in Mediterranean countries [52]. Flowering is also important since an olive grove must rely on the synchrony in the flowering dates between varieties for an effective cross-pollination [7,53]. In our case, cross-validated phases ended on flowering. However, our approach, if applied to other trees or crops, might also lead to significant results for phases ending at phenological events of low agronomic or ecological interest.

Secondly, the PHENAGRI data set was built 20 years ago. Nevertheless, we adopted it because, to our knowledge, PHENAGRI has the unique advantage of having a complete screening of olive phenology over the whole olive-growing cycle from several varieties and experimental sites scattered all over Italy (Central, South Italy, and islands). We believe that the use of more recent data is more important for the analysis involving short life cycle crops, especially if such crop varieties are subjected to continuous genetic improvement.

Thirdly, in our work, the budbreak must be known to predict flowering. Nevertheless, in absence of budbreak’s observations, the model could be used for impact analyses using scalar budbreak dates over its common time frame. Moreover, the use of time phase lengths has the unique advantage of being usable for a reverse, large-scale, modelling approach, allowing us, in our case, to trace back the budbreak dates (rarely observed) starting from the observations of flowering (commonly observed), paving the way to a better understanding of how the climate could induce plants to break the winter rest.

## 5. Conclusions

In conclusion, we presented a generalized phenological model for olive that could be used throughout Italy for several applications. Some of these may be simulations for groves with landraces or mixed varieties, regional phenological bulletins on flowering, flowering predictions for decision-making support systems, and reverse modelling to reconstruct budbreak data. Such applications may be helpful for many crop management issues, such as scheduling crop practices, reducing climate risks, optimizing external resources, and enhancing pest and disease control.

Our work showed that the linearity between phase developmental rate and temperature is of extreme relevance for the accuracy of the thermal sum model. Indeed, when there exists a strong linear temperature response it becomes possible to obtain a unique calibration of the thermal sum model even on a large-scale domain and at the species level.

Nevertheless, further investigations will be necessary to develop a predictive model for budbreak and fruit development in order to help the scientific community, decision-makers, and farmers to manage the impacts of the ongoing climate changes.

## Figures and Tables

**Table 1:** Coordinates of experimental sites and olive varieties monitored in the PHENAGRI project.

Site	Latitude	Longitude	Varieties
Montepaldi (Tuscany, FI)	43.66	11.14	Carolea, Coratina, Picholine, Frantoio, Leccino, Moraiolo, Pendolino
Villasor (Sardinia, CA)	39.38	8.91	Carolea, Coratina, Picholine, Bosana, Tonda di Cagliari
Valenzano (Apulia, BA)	41.03	16.85	Carolea, Coratina, Picholine, Nocellara Etnea
Torre Alegra (Sicily, CT)	37.41	15.00	Carolea, Coratina, Picholine, Moresca, Tonda di Iblea
BeliceMAre (Sicily, TP)	37.60	12.85	Carolea, Picholine, Biancolilla, Nocellara Etnea, Nocellara Messinese
Rende (Calabria, CS)	39.36	16.23	Carolea, Coratina, Picholine, Cassanese, Nocellara Messinese
Prepo (Umbria, PG)	42.99	12.26	Carolea, Coratina, Picholine, Frantoio, Moraiolo

**Table 2:** Observed phenological events for *Olea europaea* under the PHENAGRI project.

BBCH Scale	Description
01	Foliar buds start to swell and open
03	Foliar buds lengthen and separate from base
07	External small leaves open, not completely separated
11	First leaves completely separated
50	Inflorescence buds leaf axils completely closed
51	Inflorescence buds start to swell
55	Flower cluster totally expanded
61	Beginning of flowering
65	Full flowering, at least 50% of flowers open
68	Majority of petals fallen or faded
69	End of flowering, non-fertilized ovaries fallen
71	Fruits at 10% of final size
75	Fruits at 50% of final size
80	Fruit becoming light green or yellowish
81	Beginning of fruit coloring
85	Increasing specific fruit coloring
89	Harvest maturity
92	Overripe with fruits that start to fall
99	At least 50% of fruits fallen

**Table 3:** Statistical evaluation of simple *DR*s (Equation (4)) obtained from the KfCV for selected phases. S * = mean observed phase time length; RMSE = Root Mean Square Error [d]; *r*^2^ = coefficient of determination (* = adjusted *r*^2^); MBE = Mean Bias Error [d].

From BBCH	To BBCH		*r* ^2^	*p*-Value	RMSE [days]	MSE [days]	S * (St. Dev) [days]	N. Sites	N. Data
01	61	Model of Equation (1)	0.95	2.0 × 10^−5^	4.7	−0.3	64 (22)	6	50
		Model of Equation (2)	0.96 *	2.7 × 10^−6^	4.3	−0.8			
		Model of Equation (3)	0.93	6.1 × 10^−4^	6.4	−3.2			
07	61	Model of Equation (1)	0.93	3.6 × 10^−7^	5.6	−0.5	60 (22)	7	71
		Model of Equation (2)	0.93 *	2.9 × 10^−7^	5.6	−0.59			
		Model of Equation (3)	0.92	9.8 × 10^−7^	7.1	−3.1			
51	61	Model of Equation (1)	0.93	1.2 × 10^−8^	6.6	−2.0	37 (22)	7	84
		Model of Equation (2)	0.89 *	2.4 × 10^−7^	6.9	−1.1			
		Model of Equation (3)	0.91	1.5 × 10^−7^	7.3	−2.9			
01	65	Model of Equation (1)	0.96	7.7 × 10^−7^	3.9	−0.1	71 (21)	6	51
		Model of Equation (2)	0.98 *	1.6 × 10^−7^	3.5	−0.2			
		Model of Equation (3)	0.93	7.4 × 10^−6^	6.8	−3.7			
07	65	Model of Equation (1)	0.93	4.6 × 10^−7^	5.6	−0.5	68 (22)	7	72
		Model of Equation (2)	0.93 *	5.6 × 10^−7^	5.6	−0.3			
		Model of Equation (3)	0.92	2.9 × 10^−7^	8.1	−4.6			
51	65	Model of Equation (1)	0.93	1.8 × 10^−7^	5.7	−1.0	44 (21)	7	85
		Model of Equation (2)	0.92 *	2.0 × 10^−7^	5.8	−0.7			
		Model of Equation (3)	0.86	1.2 × 10^−5^	14.8	−5.4			

**Table 4:** Final calibration of linear *DR*s for cross-validated phases. *T*_0_ = base temperature (see SSection 2.2.1).

From BBCH	To BBCH	*r* ^2^	*p*-Value	*DR = a + bT*		
				*a*	*b*	*T*_0_ * = −a/b*
01	61	0.85	1.8 × 10^−21^	−0.0180	0.0025	7.2
07	61	0.79	1.1 × 10^−25^	−0.0253	0.0031	8.2
51	61	0.62	5.9 × 10^−19^	−0.0817	0.0075	10.9
01	65	0.89	1.2 × 10^−25^	−0.0132	0.0020	6.7
07	65	0.81	1.7 × 10^−27^	−0.0187	0.0024	7.8
51	65	0.74	2.9 × 10^−^^26^	−0.0540	0.0050	10.7

## Data Availability

The data presented in this study are available on request from the corresponding author.

## Supplementary Materials

The following are available online at https://www.mdpi.com/article/10.3390/plants10061115/s1. Figure S1: Examples of how a linear DR may fit the data over three phases (reported in the legend), Figure S2: As Figure S1 but for multiple linear regressions between developmental rates, air temperature, and photoperiod, Figure S3: As Figure S1 but for polynomial regression between developmental rates and air temperature, Figure S4: Example of training and validation within the KfCV, Figure S5: Developmental rates vs. temperature for the cross-validated phases from data embracing the olive varieties common to all locations, Figure S6: Correlation matrix between time phase length, temperature, and precipitation.
