# Supplementary material for: A Phenological Model for Olive (Olea europaea L. var europaea) Growing in Italy
Source: Plants (Basel). 2021 May 31;10(6):1115. doi: 10.3390/plants10061115 (PMC8230019; doi:10.3390/plants10061115)
Supplement: Supplementary file 1 [file plants-10-01115-s001.zip › plants-1181817-supplementary.pdf]

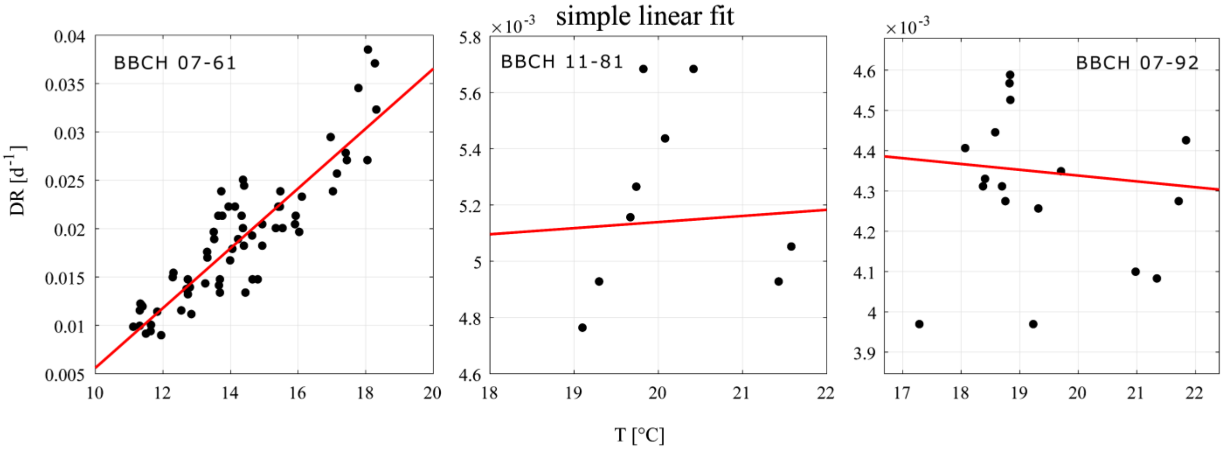

Figure S1. Examples of how a linear DR may fit the data over three phases (reported in each plot). The panels show how a simple linear regression (red line) could result over phases showing a likely linear relation (left panel), a non-linear relation (central panel) and no relation at all (right panel) between developmental rates and air temperature. Phases reported here are defined in Table 2 of the main text.

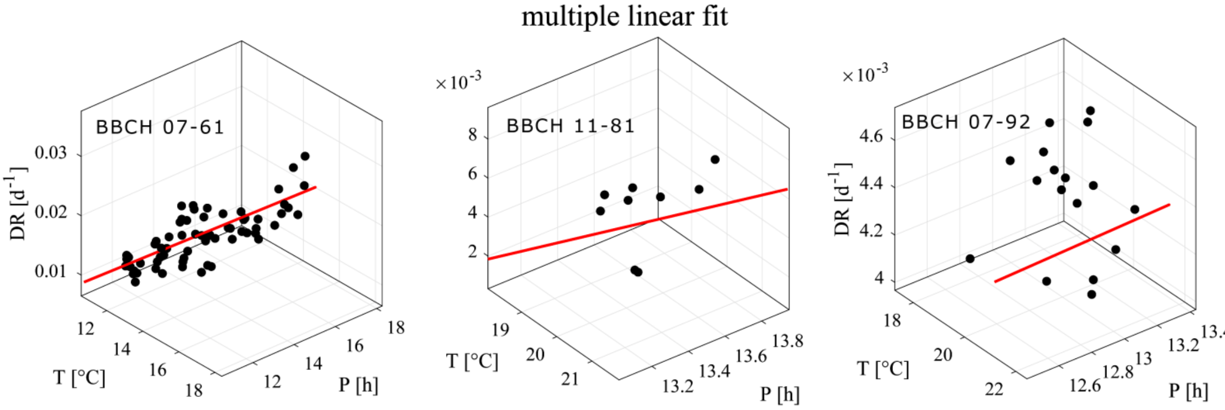

Figure S2. As Figure S1 but for multiple linear regression between developmental rates, air temperature, and photoperiod.

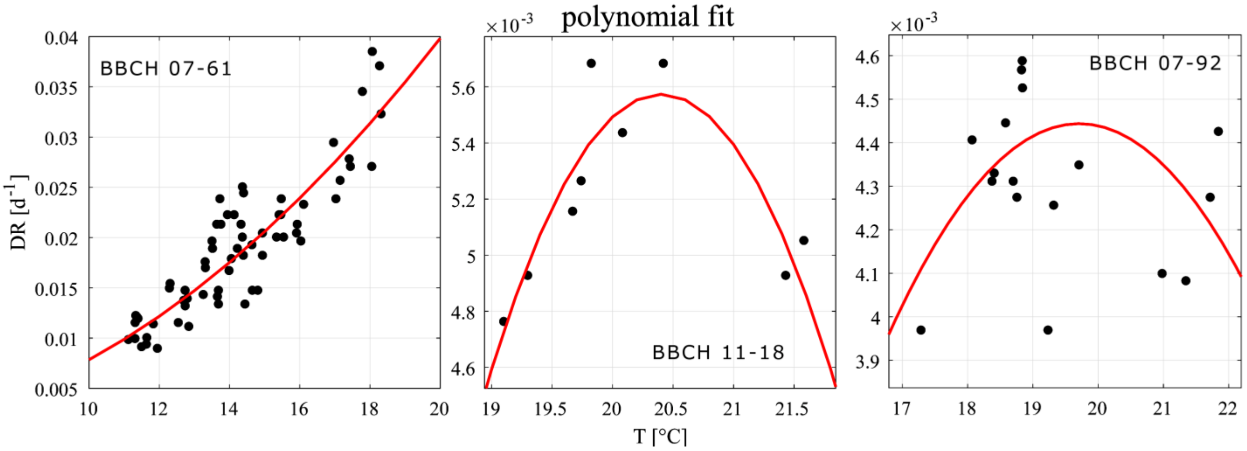

Figure S3. As Figure S1 but for polynomial regression between developmental rates and air temperature.

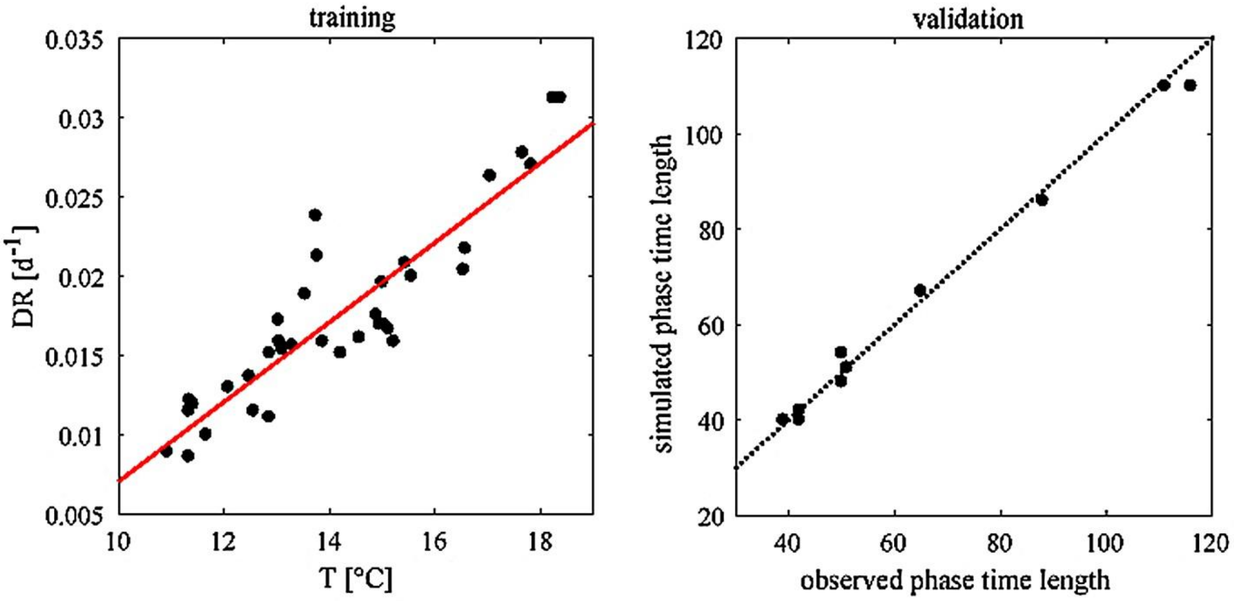

Figure S4. Example of training and validation within the KfCV for the phase BBCH 01-61. In the training procedure the developmental rates are regressed against mean air temperature using the training subset (K−1 folds). In the validation procedure the regression is used to simulate the phase time length over the validation subset (k-fold). Left panel: training; right panel: validation; red line: least-square line in the form of eq. (1); dotted black line: 1:1 line. In this example k = 1, n = 1, a = −0.0181 d^{-1}; b = 0.0025 °C^{-1}d^{-1}, T0 = 7.2 °C; r^2 = 0.99; RMSE = 2.6 days.

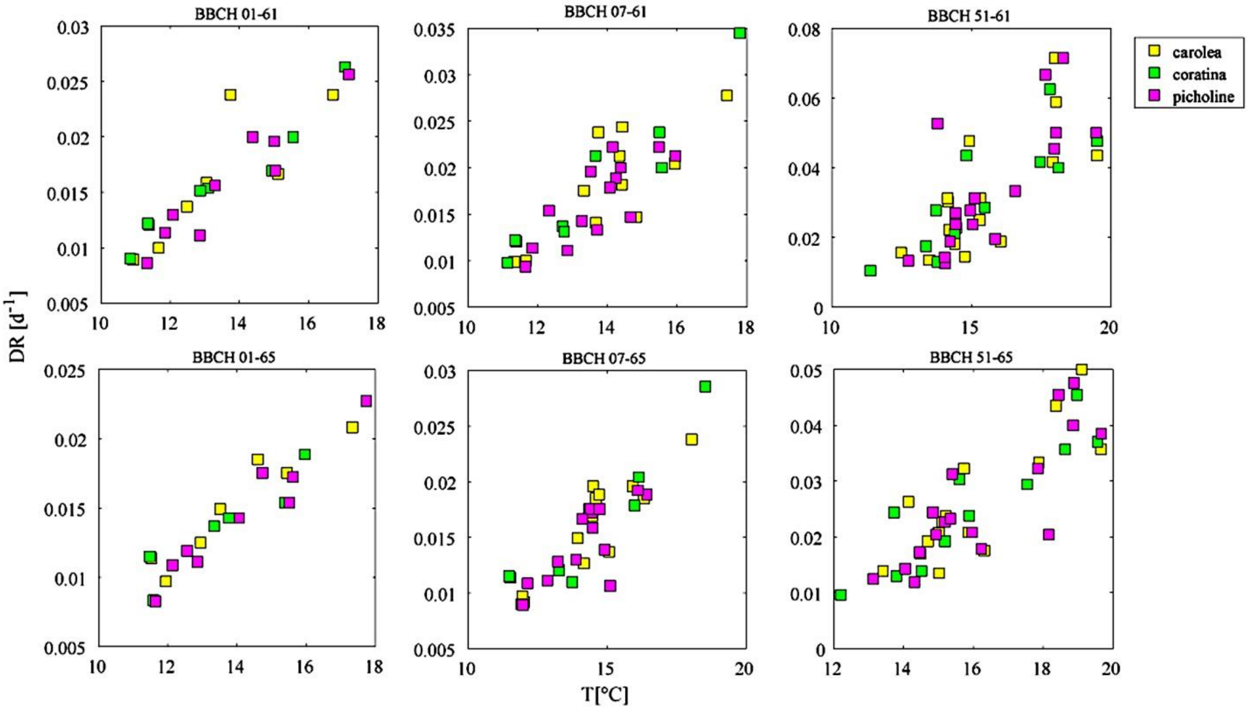

Figure S5. Developmental rates vs. temperature for the cross-validated phases (subplots headline) from data embracing the olive varieties common to all locations (Table 1 in the main text). Phases reported here are defined in Table 2 of the main text. The different olive varieties are presented in different colours.

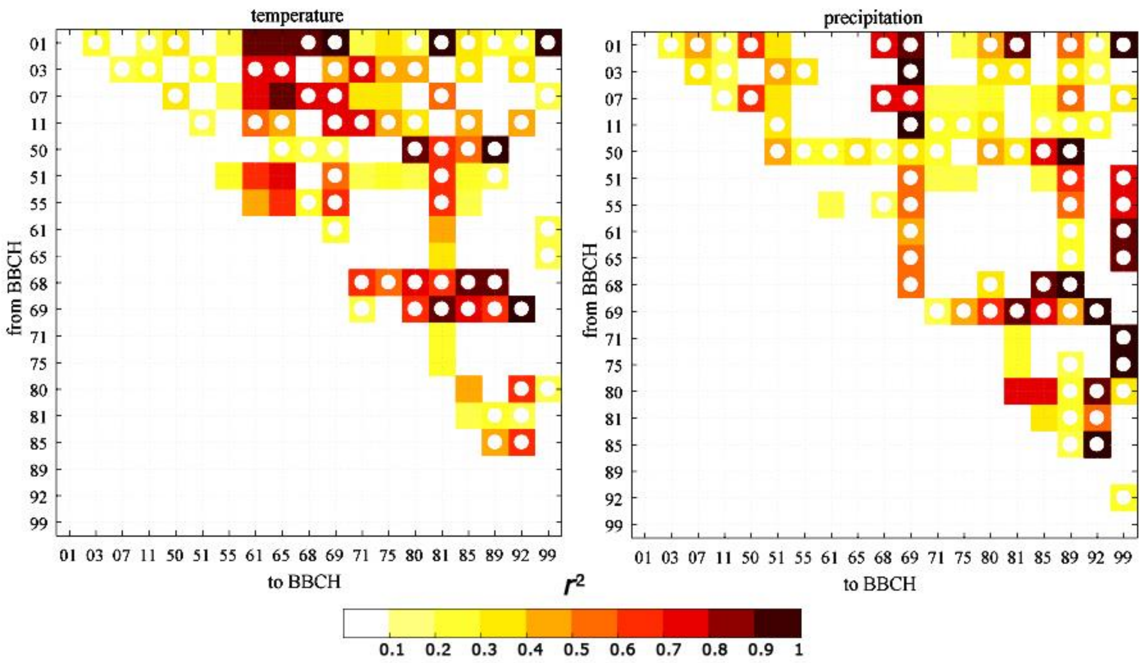

Figure S6. Correlation matrix between phase time length and temperature (left panel) and cumulated precipitation (right panel). x-axis: phenological events defining the beginning of a phase, expressed in BBCH scale (definition in Table 2); y-axis: phenological events endings the phase, expressed in BBCH scale. Empty cells indicate the absence of data, white dot markers indicate phases discarded because of data embrace less than 5 experimental sites.
